# Structure of Sodium Carboxymethyl Cellulose Aqueous Solutions: A SANS and Rheology Study

**DOI:** 10.1002/polb.23657

**Published:** 2014-12-30

**Authors:** Carlos G Lopez, Sarah E Rogers, Ralph H Colby, Peter Graham, João T Cabral

**Affiliations:** 1 Department of Chemical Engineering, Imperial College LondonLondon, SW7 2AZ, United Kingdom; 2 Diffraction and Materials Division, ISIS-STFC, Rutherford Appleton Laboratory, Chilton, Oxon OX11 0QXUnited Kingdom; 3 Department of Materials Science and Engineering, The Pennsylvania State University, University ParkPennsylvania, 16802; 4 Unilever Research Port Sunlight LaboratoryQuarry Road East, Bebington L63 3JW, United Kingdom

**Keywords:** polyelectrolytes, cellulose, neutron scattering, rheology, structural characterization, water-soluble polymers

## Abstract

We report a small angle neutron scattering (SANS) and rheology study of cellulose derivative polyelectrolyte sodium carboxymethyl cellulose with a degree of substitution of 1.2. Using SANS, we establish that this polymer is molecularly dissolved in water with a locally stiff conformation with a stretching parameter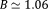. We determine the cross sectional radius of the chain (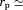 3.4 Å) and the scaling of the correlation length with concentration (*ξ* = 296 *c*^−1/2^Å for *c* in g/L) is found to remain unchanged from the semidilute to concentrated crossover as identified by rheology. Viscosity measurements are found to be in qualitative agreement with scaling theory predictions for flexible polyelectrolytes exhibiting semidilute unentangled and entangled regimes, followed by what appears to be a crossover to neutral polymer concentration dependence of viscosity at high concentrations. Yet those higher concentrations, in the concentrated regime defined by rheology, still exhibit a peak in the scattering function that indicates a correlation length that continues to scale as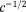. © 2014 The Authors. Journal of Polymer Science Part B: Polymer Physics Published by Wiley Periodicals, Inc. J. Polym. Sci., Part B: Polym. Phys. **2015**, *53*, 492–501

## INTRODUCTION

### Cellulose and Cellulose Derivatives

Cellulose is the most abundant polymer on Earth with annual cellulose synthesis by plants approaching 10^12^ tons.^1^ Although cellulose is insoluble in water and most common organic solvents,^2^ cellulose derivatives are soluble in a wide range of solvents;^3^ for example, cellulose acetate is soluble in acetone, tetrahydrofuran, and other organic solvents while methylcellulose, ethyl hydroxyethyl cellulose, and sodium carboxymethyl cellulose (NaCMC) are water soluble.^2^,^4^ Cellulose derivatives can be dissolved either at a molecular or colloidal level.^2^,^3^

**Figure 1:**
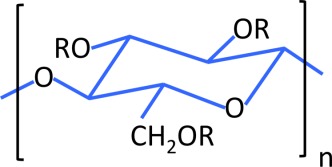
Monomer structure of sodium NaCMC, where R can be H or CH_2_COONa. The degree of substitution (D.S.) is defined as the average number of CH_2_COONa groups per monomer out of a maximum of 3. [Color figure can be viewed in the online issue, which is available at http://wileyonlinelibrary.com.]

Carboxymethylcellulose (CMC), generally used as sodium salt NaCMC, is one of the most widely used polyelectrolyte cellulose derivatives.^5^ NaCMC is an anionic, water soluble, polyelectrolyte with vast applications in the food, pharmaceutical, personal care/cosmetic, paper and other industries.^4^ For instance, NaCMC is extensively used as a rheology modifier in common toothpastes and shampoos,^6^ as a film former in textile treatments^7^ and to prevent the redeposition of soil removed by detergents during the fabric washing process.^8^ Its monomer structure is shown in Figure 1, where R stands for –H or –CH_2_CO_2_Na. The degree of substitution (D.S.) is the average number of carboxymethyl groups substituted per monomer unit, ranging from 0 to 3 with the remaining R = H.

Aqueous solutions of NaCMC are generally complex. Some studies suggest that only a fraction is molecularly dispersed,^9^,^10^ implying that NaCMC is a colloidally dispersed polymer while others report full solubility in dilute salt solutions.^11^,^12^ The degree of solubility is accepted to be a function of the degree of substitution, with less substituted (more hydrophobic) NaCMC showing a larger fraction of aggregates.^10^ Aggregated crystalline domains have been found by X-ray diffraction in aqueous solution,^13^ with degree of crystallinity correlating with lower D.S. and no crystallinity found for D.S.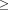 1.06. Additionally, low substitution grades often exhibit thixotropy^10^,^14^ which is thought to result from the formation of networks or soft aggregates.^10^ The regularity of monomer substitution, instead of the average D.S., was found to be the main factor controlling the rheological properties of NaCMC aqueous solutions.^10^ NaCMC of low D.S. but uniform substitution, that is, lacking blocks of unsubstituted monomers, yields similar properties to highly substituted grades. Typically, samples with D.S.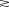 1, do not exhibit thixotropy as, with increasing D.S., substitution becomes more random. For this study, we have selected a sample of D.S.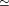 1.2 and, therefore, expect no significant blocks of unsubstituted cellulose, and thus no thixotropy or gelling at high concentrations. According to a model by Reuben et al.,^15^,^16^ for an average D.S. = 1.2, 10–20% of the monomers will not be substituted and thus, even in the absence of large unsubstituted blocks, hydrophobic interactions from unsubstituted monomers remain. A range of intrinsic persistence lengths (*l*_0_) for NaCMC have been reported, varying from 5 to 15 nm^11^,^17^,^18^ characteristic of a semiflexible polymer and typical of many polysaccharides.^18^,^19^

The rheology of NaCMC in aqueous, salt free, solutions has been previously studied.^5^,^10^,^14^,^20^–^24^ A Fuoss or near Fuoss dependence of the specific viscosity (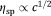) has been reported in the semidilute unentangled regime.^20^–^22^,^24^ A strong dependence of the specific viscosity (*η*_sp_) with polymer concentration (*η*_sp_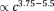) is reported at high concentration.^20^,^24^

Simulations by Wang and Somasundaran^25^ suggest that NaCMC adopts a helical conformation in bulk and in solution while Biermann et al.^26^ found that, depending on the substitution pattern, the chain may be locally collapsed or highly extended. Although a helical chain conformation is suggested by optical rotary dispersion and circular dichroism data,^27^–^29^ light scattering, intrinsic viscosity, potentiometric titration, and dielectric spectroscopy data have been successfully interpreted assuming a locally linear conformation.^11^,^17^,^24^,^30^,^31^

Significant questions regarding the local conformation and solubility of NaCMC in water thus remain unresolved. In this work, we use small angle neutron scattering (SANS) to probe the solubility and conformation of NaCMC in aqueous solution. We complement SANS results with careful steady shear rheological measurements across a wide concentration range, which allows us to deduce the different concentration regimes and obtain further insight into the conformation and dynamics of NaCMC in water.

## BACKGROUND THEORY

### Scaling Theory

The Bjerrum length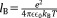, where *e* is the unit of charge, *ε*_0_ is the vacuum permitivity, *ε* is the relative dielectric constant, and *k*_B_ is the Boltzmann constant, is defined as the distance at which the electrostatic energy between two charges is equal to the thermal energy (7.1 Å for water at 25

C). Salt free solutions of flexible polyelectrolytes can be classified into dilute, semidilute (non entangled and entangled), and concentrated. In dilute solutions, *c* < c*, where c* is the overlap concentration, polyelectrolytes adopt an extended conformation and can be represented by a directed random walk of electrostatic blobs of diameter *ξ_T_*.


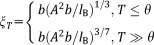
(1)

where *A* is the number of monomers per effectively charged group, *θ* is the theta temperature, and *b* is the monomer length. For distances below *ξ_T_*, thermal energy dominates and the chain conformation depends on the solvent quality, becoming extended in good solvent and collapsed in poor solvent. The stretching parameter (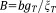), where *g_T_* is the number of monomers in an electrostatic blob, is defined as the ratio between the polymer contour length *L* = *Nb*, where *N* is the degree of polymerization, and the end-to-end length in dilute solution, *R*_ee_ = 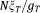. The scaling prediction for the specific viscosity at zero shear rate (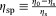, where *η*_0_ is the dynamic viscosity at zero shear rate and *η*_s_ is the solvent's dynamic viscosity) in dilute solution is *η*_sp_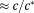. Throughout this article, we use *η*_sp_ to refer to the specific viscosity in the Newtonian (zero shear rate) limit.

At c*, estimated by


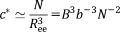
(2)

chains begin to overlap. In the semidilute regime, the most relevant length scale is now the correlation length *ξ*; at length scales smaller than *ξ*, chains do not interact and the conformation is like that of a dilute solution; at length scales larger than *ξ*, the electrostatic energy is screened and chains follow random coil statistics. A peak in the structure factor, *S*(*q*), is observed in flexible semidilute salt free polyelectrolytes, whose position scales as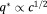, in agreement with the scaling prediction:


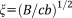
(3)

Wavenumber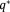 can be related to the correlation length, *ξ*, by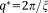. The origin of this peak has been assigned to the inability of correlation blobs to overlap due to the strong repulsion generated by the counterion clouds surrounding the polyions, thereby resulting in the suppression of long range fluctuations.^32^,^33^ This is also manifested in the unusually low compressibility of polyelectrolyte solutions.^34^

In the semidilute unentangled regime, the specific viscosity of salt free polyelectrolyte solutions is given by:


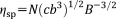
(4)

This power law dependence with *c* is usually referred to as the Fuoss law,^35^ which has been observed for a number of polyelectrolyte systems, although its validity has been questioned.^36^,^37^ Equation 4 predicts the viscosity to depend linearly on *N* while, experimentally, it has been found that *η*_sp_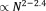 for sodium polystyrene sulfonate (NaPSS),^37^ thus suggesting the scaling argument might not be valid. However, the concentration dependences of both the viscosity and longest relaxation time^38^,^39^ have been found experimentally and via simulations^40^ to agree with those expected from the Rouse model, as has the experimentally measured relaxation time distribution.^41^

Chains start to entangle at *c*_e_ = *n*^4^c*, where *n* is the number of overlapping chains necessary to form an entanglement. The tube diameter can be calculated to be *a* = 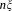 and the reptation time and viscosity in semidilute entangled solutions are given by:


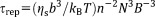
(5)


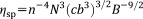
(6)

The crossover concentration to the concentrated regime (*c*_D_) is expected when the correlation blob reaches the electrostatic blob, that is *ξ_T_* = *ξ*. The chains are then predicted to behave similarly to neutral polymers and the viscosity varies as *η*_sp_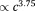 in good solvent and *η*_sp_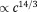 in *θ* solvents.^42^ These high exponents for the concentration dependence of the specific viscosity have been experimentally observed.^38^,^43^ For polyelectrolytes in poor solvent, there is no predicted exponent but an even stronger concentration dependence may be expected as counterions condense to form ion pairs that attract each other. The terminal modulus is expected to scale as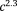 for neutral polymers in good or *θ* solvent. This scaling is, however, not observed for concentrated polyelectrolyte solutions,^38^,^43^ which are therefore rheologically distinct from neutral polymers. Scaling theory does not give a prediction for the existence of a peak in the scattering function in this regime. Experimentally, it has been observed that for NaPSS, the scaling of the peak position with concentration changes from 0.5 to 0.25, coinciding with a change in the scaling of osmotic pressure with concentration, approaching a value similar to that of neutral polymers.^34^ A crossover to the 0.25 exponent has also been reported for less flexible polyelectrolytes poly(*α*-methylstyrene sulfonate), poly(diallyl dimethylammonium chloride), and hyaluronic acid at intermediate concentrations, followed by a steeper power law of *c*^1^ at higher concentrations for the latter two.^44^,^45^

### Scattering

We next consider the analysis and interpretation of SANS data of NaCMC solutions in the semidilute and concentrated regimes. The form factor of polyelectrolytes in solution can be described by a wormlike chain model. At high *q*, where we expect no intermolecular effects,^46^,^47^ the data are fitted with:


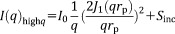
(7)

The first term models the scattering of a wormlike chain with a cross sectional radius *r*_p_. The constant *I*_0_ is a function of the contrast factor, the polymer concentration, and the monomer length (detailed in Supporting Information); *J*_1_ is a first-order Bessel function of the first kind, and the term *S*_inc_ accounts for the *q*-independent incoherent scattering. The (intermolecular) structure factor of polyelectrolyte solutions is complex to model^48^ and we, therefore, use an empirical approach by multiplying eq 7 by


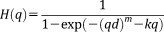
(8)

which yields a descriptive fit to the data from which the peak position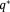, peak intensity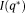, and a sharpness parameter can be extracted. We assign no physical meaning to the fitting parameters *m*, *d*, and *k*. Polyelectrolyte solutions exhibit a scattering upturn at low *q* values, with power laws between 2 and 4^49^–^55^ which are not expected from scaling theory^32^,^33^ or PRISM.^48^ The origin of this upturn remains controversial,^49^,^56^,^57^ as it appears incompatible with the suppression of long range concentration fluctuations by the large osmotic pressure created by counterions, which serves as a definition of the correlation length in scaling theory.^33^ To complete our fitting procedure, we include an additive power law term to eqs 7 and 8,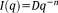 and determine *D* and *n* as a function of concentration. A detailed account of this fitting procedure is provided in Supporting Information.

## EXPERIMENTS

NaCMC of average degree of substitution (D.S.) 1.15–1.45 and nominal *M*_w_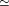 250 kg/mol was purchased from Sigma Aldrich. Aqueous GPC with elution solvent 0.3 M NaNO_3_/0.002 M NaOH yields *M*_w_ = 280 kg/mol and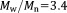. The degree of substitution was measured by converting the polymer into its acid form in a nitric acid-ethanol mixture, adding a known amount NaOH and titrating the excess NaOH with HCl using phenolphthalein as an indicator, giving D.S. = 1.17 ± 0.10. The residual salt in the polymer was calculated by dialyzing a solution against DI water (using a 10 kDa Spectrapor membrane) and measuring the conductivity increase of the water. Using a totally dissolved solids conversion factor of 0.6 µS/pmm, the salt content in the polymer is estimated to be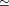 0.4 wt %, in good agreement with the typical purity of NaCMC samples (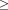
99.5 wt %). Water was obtained from a MilliQ source and with resistivity of 18 MΩ/cm. D_2_O (99.8% D content) was purchased from Cambridge Isotopes. Rheological measurements were made on a stress controlled TA hybrid rheometer using a cone and plate geometry of diameter 40 or 60 mm and angle of 1 or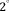, as well as an Anton Paar Physica MCR 301 using a double gap geometry, with internal and external gaps of 0.5 mm.

SANS experiments were carried out on three diffractometers: time-of-flight SANS2D (ISIS, STFC, HSIC, Didcot, U.K.) with *q*-range of 0.0045–0.8 Å^−1^; D11 (ILL, Grenoble, France) with incident *λ* = 6 Å and sample-detector distances of 1.2 m, 8 m, 39 m, yielding *q*-range 0.0013–0.5 Å^−1^; and D22 (ILL, Grenoble, France) with *λ*= 6 Å and sample-detector distances of 1.5 m, 5.6 m, 17.6 m yielding 0.003–0.6 Å^−1^. Quartz Hellma cells of path length 5 or 2 mm were used, depending on polymer concentration.

## RESULTS

### Rheology

**Figure 2:**
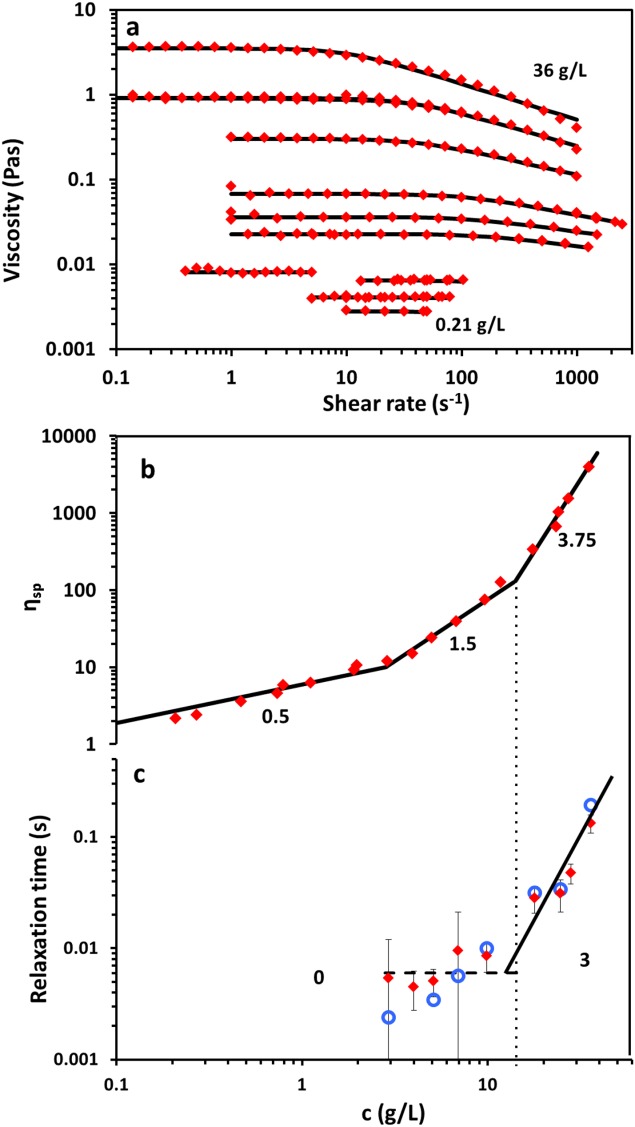
Rheology of NaCMC aqueous solutions. (a) Representative rheograms for *c* = 0.21 to 36 g/L, (b) specific viscosity as a function of concentration. The lines indicate the scaling predictions for the semidilute unentangled, semidilute entangled and concentrated power laws. c) Relaxation times (*τ*) as a function of concentration, red ♦ from Carreau model and blue • from shear rate at which viscosity is equal to 90% of its zero shear value. Lines indicate power laws of 0 and 3. The dotted vertical line indicates c_D_ = 14.4g/L. [Color figure can be viewed in the online issue, which is available at http://wileyonlinelibrary.com.]

Figure 2 summarizes representative results of NaCMC solutions. We first obtain the zero shear rate viscosity (*η*_0_) from the Newtonian region of the profile and then fit the viscosity dependence of the shear rate to the Carreau model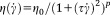, where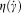 is the dynamic viscosity of the solution, *τ* [s] is a constant, which may be identified with the inverse of the shear rate at which shear thinning starts, *p* is an exponent which determines the power law of the viscosity with shear rate in the shear thinning regime and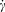 [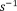] is the shear rate. We also estimate the relaxation time from the inverse shear rate at which the viscosity reaches 90% of its zero shear rate value. For samples with *η*_sp_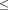 10, inertial effects become important at high shear rates which lead to an apparent increase in the viscosity. We, therefore, consider only the Newtonian region below 100 s^−1^, corresponding to the onset of inertial instabilities in water, and extract its Newtonian viscosity value. Figure 2(b) shows the specific viscosities (*η*_sp_) of NaCMC solutions as a function of concentration. The lines are the scaling predictions for the slopes of *η*_sp_ for the semidilute unentangled (0.5), entangled (1.5) and concentrated regimes (3.75). These compare well to the best fit slopes 0.68 ± 0.02, 1.7 ± 0.2, and 3.4 ± 0.3, respectively, and we identify *c*_e_ to be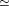 2.9 g/L and *c*_D_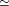 14.4 g/L using the scaling theory power laws and *c*_e_ = 4 g/L and *c*_D_ = 14.3 g/L using the best fit power laws. Although our measurements do not extend to low enough concentrations to measure the overlap concentration c*, we can obtain an estimate by extrapolating the unentangled data (using the best fit 0.68 exponent) to *η*_sp_ = 1, which gives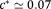 g/L. The relaxation times in the 3–14 g/L range, within the semidilute entangled region as identified from viscosity, remain constant within experimental error, in agreement with eq 4. The relaxation times for concentrations above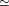 14 g/L vary as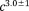, which is higher than the scaling prediction for entangled neutral polymers in both good (1.6) and *θ* (2.3) solvents, but the uncertainty is large as the concentration is only varied by a factor of ≃ 2 in this concentration regime.

We tested a high concentration (*c* = 40 g/L) sample for thixotropy by measuring its viscosity in the shear rate range of 1 to 800 s^−1^ before and after shearing for 2 min at 800 s^−1^. No time dependence of the viscosity was observed, and we, therefore, assume that all the samples studied (*c*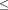 40 g/L) are nonthixotropic.

### Small angle neutron scattering

**Figure 3:**
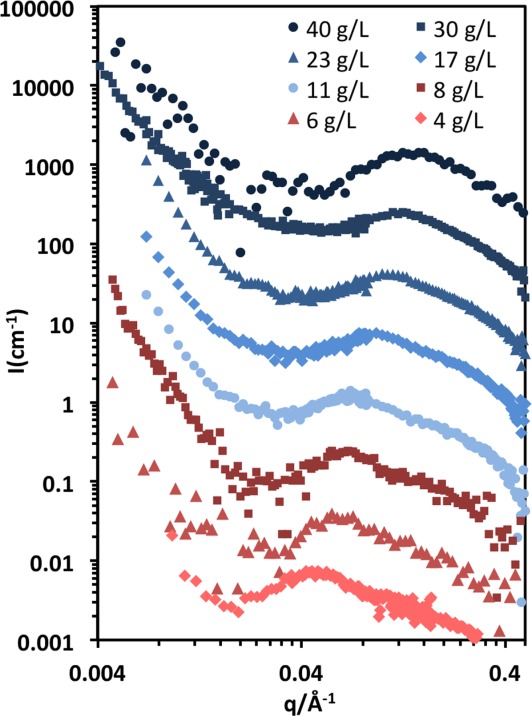
Coherent SANS profiles for aqueous solutions of NaCMC for different concentrations ranging from 4 to 40 g/L, curves shifted in 5× increments for clarity. The 4 g/L curve is not shifted. [Color figure can be viewed in the online issue, which is available at http://wileyonlinelibrary.com.]

Figure 3 shows the scattering profiles for NaCMC solutions with concentrations from 4 to 40 g/L. Typical polyelectrolyte features are observed, namely the presence of a correlation peak, whose position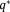 increases with concentration, and the characteristic low *q* upturn discussed earlier.

#### High q: The Chain Diameter

**Figure 4:**
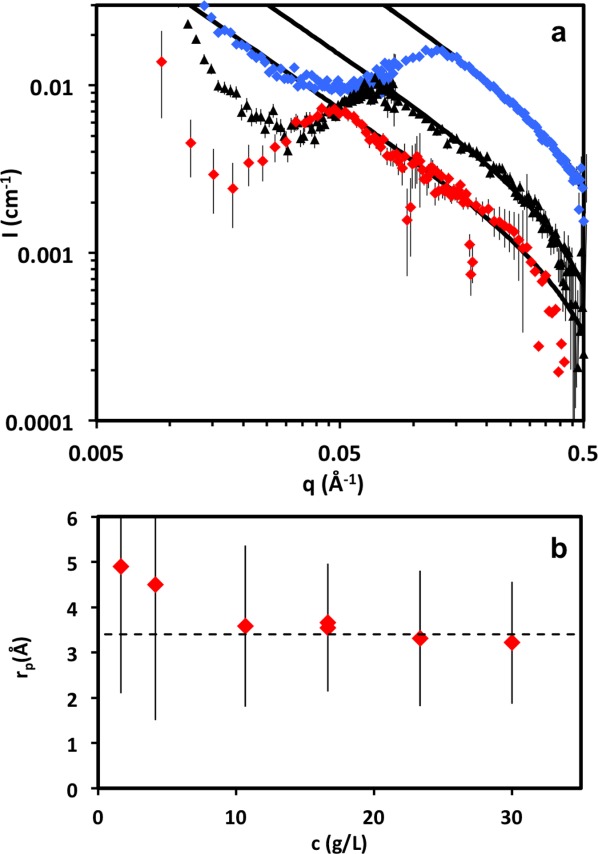
(a) Coherent SANS profiles for NaCMC solutions with concentrations: Blue ♦ *c* = 30 g/L, black ▴ *c* = 11 g/L, and red ♦ *c* = 4 g/L. Black line: fit to *q* > 1.5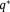 data using eq 7. (b) Cylindrical chain radius as a function of concentration. Dashed line indicates *r*_p_ = 3.4 Å. [Color figure can be viewed in the online issue, which is available at http://wileyonlinelibrary.com.]

The D11 and D22 profiles were fitted with eq 7. We find that more consistent results are obtained when fitting the range *q* > 1.5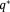, and representative fits are shown in Figure 4(a). A Gaussian, instead of step density, chain cross section profile gives similar values of *r*_p_. An alternative fitting procedure using a helical form factor,^58^ described in Supporting Information, yields an effective chain radius of 3.0 ± 1.5 Å and linear mass density of the chain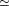 5% higher than the linear chain, such that these two descriptions are effectively very similar. We adopt the wormlike model throughout the article and comment on the (minor) differences for the helical case where appropriate. The wormlike chain model describes the higher concentrations well but becomes poorer for *c*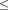 10 g/L, when an additional feature, a weak broad shoulder appears at high *q*. This feature has been observed in dilute solutions of NaPSS^59^ and semidilute solutions of tobacco mosaic virus,^60^ as well as in the small angle X-ray scattering (SAXS) of sodium alginate (in the concentration range of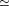 2–6g/L, although not discussed).^61^

More complex form factors, including the pearl necklace, elliptical cross section cylinder, two cylinders of different cross sections, the fraction and radii of which were left as free parameters, and single or double helix (pitch with an upper bound of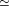 200 Å) do not yield significantly better agreement than a single cylinder. Including the contribution of the counterions, as calculated by the Katchalsky cell model,^47^,^62^,^63^ also did not improve the fit.

The *r*_p_ values obtained from fitting the data with eq 7 are plotted in Figure 4(b). The slight increase at low concentrations is possibly an artefact due to the scattering shoulder at lower concentrations. The values are approximately constant for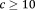 g/L.

A value of *r*_p_ = 3.4 Å, indicated by the dashed line in Figure 4(b) is selected and imposed on subsequent data fits for all the samples, with *I*_0_ and *S*_inc_ now being the only free parameters. The model agreement with the data does not change significantly compared to leaving *r*_p_ as a free parameter. To ensure consistency, we evaluate the dependence of *I*_0_ and *S*_inc_ with concentration, as we expect *S*_inc_ to be proportional to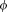, the volume fraction occupied by monomers, and *I*_0_ to be proportional to
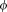
(1 − 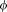), both with intercept at the origin. This is confirmed with a linear regression correlation coefficient (*R*^2^) in the range of 0.99 to 0.93 for all datasets. Comparing the value of *I*_0_ with the calculated one from the SLDs and partial molar volume of CMC,^64^ we estimate the water content in the samples to be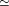 14% for SANS 2D and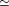 19% for the D11 and D22 samples, the difference arising possibly due to slightly different drying conditions and small errors in the absolute calibration of the neutron data. All the concentrations in this article are reported using the corrected concentration (assuming the water content to be 18% for the rheology samples). The incoherent scattering of the polymer is found to be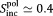 ± 0.1 cm^−1^.

#### Intermediate and Low q-Ranges: Correlation Peak and Upturn

**Figure 5:**
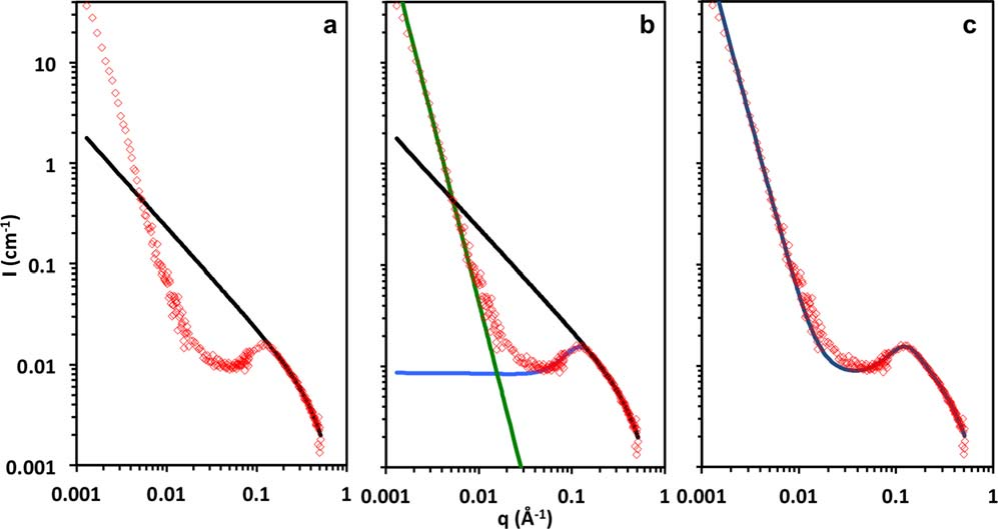
Sequence of fitting stages for 30 g/L aqueous solution of NaCMC. (a) Wormlike chain fit to high *q* data, shown in black; (b) the peak function (blue line) and the upturn, modeled as a power law fit (green line); (c) the overall fit to the data. [Color figure can be viewed in the online issue, which is available at http://wileyonlinelibrary.com.]

Figure 5 shows the sequential stages of data fitting. First, a wormlike chain form factor with finite cross section, determined previously, is fitted to the high *q* data, indicated by the black line. Then, we use eq 8 to fit the correlation peak (blue line), and finally, we add an upturn term D/*q^n^* indicated by the green line. We find that *n* = 3.6 is close to the best fit slope for all samples and is thus fixed.

**Figure 6:**
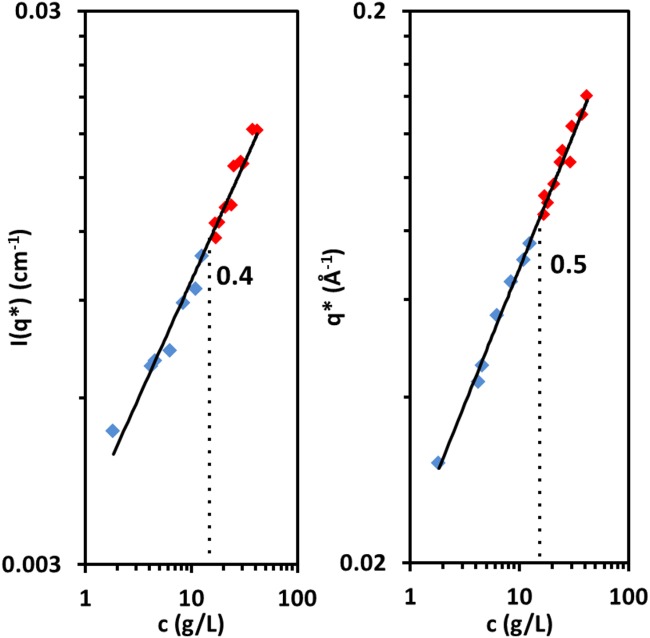
Correlation peak intensity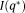 and position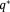 as functions of concentration; blue symbols for samples in the semidilute regime and red for those in the concentrated. Numbers indicate the slopes for the best fit single power law. The dotted lines indicate the c_D_ as identified by rheology. [Color figure can be viewed in the online issue, which is available at http://wileyonlinelibrary.com.]

The peak position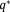 and the intensity at the peak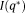 are plotted as functions of concentration in Figure 6. The best fit imposing a 0.5 power law is found for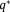 = (0.021 ± 0.003) *c*^1/2^ Å^−1^. No change in the scaling of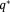 with concentration is observed across *c*_D_, the concentrated crossover, (indicated by the change of blue to red data points). The best fit power law for all samples is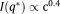, similar to what has been observed for other systems in the semidilute regime and in reasonable agreement with the scaling prediction of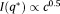. Alternatively,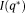 can be fitted with a 0.5 power law for
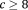
g/L and a 0.3 slope for
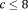
g/L. A 12 g/L sample was run at 5, 25, 45 and 65

C, and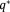 was found to be insensitive to temperature within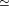 10% uncertainty.

Determining the peak width, for example as a full width half maximum parameter, is complex as it requires extrapolation into the region where the low *q* upturn dominates. We, therefore, compute the full width at 75% of its maximum value (i.e. 3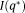/4, termed FW75M) as a measure of peak width, which yields consistent results. The peak sharpness, defined as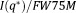 shows no trend with concentration and is approximately constant at a value of (9.6 ± 0.5) 10^8^ (dimensionless). The SANS2D data show slightly sharper peaks than those from D11 and D22, likely due to data reduction and/or a small presence of ions in the D_2_O. The dependence of the low *q* upturn intensity as a function of concentration is presented in Supporting Information; broadly we find that it increases with concentration for *c*
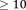
g/L.

## DISCUSSION

### Structure

#### Chain Diameter

The local conformation of a semiflexible polyelectrolyte is expected to be straight and a fit to a cylindrical form factor thus yields the cross sectional radius of the chain. The fitted radius of *r*_p_ = 3.4 ± 1.0 Å is lower than the one calculated from bond lengths 5 Å^17^ but higher than that found for sodium alginate,^65^ another polysaccharide, from SAXS. The mass per unit length and chain diameter from a fit to a single helix yields very similar results to the cylindrical fit indicating that, if a helical conformation is present, its effect on the local conformation is minor. The weak shoulder appearing in the lower concentration samples (*c*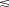 15 g/L) could not be fitted to any of the models we attempted. It has been predicted by theory^48^ and verified by experiment,^50^,^60^ that the intermolecular structure factor is not exactly equal to one at high *q* but shows small, damped oscillations, which become less prominent with increasing concentration. It is thus possible that it arises from an intermolecular effect.

#### Correlation Length

Assuming *B* = 1 (chain is rigid up to *ξ*), scaling theory predicts *ξ* = 286 *c*^−1/2^, close to the measured value of 296 *c*^−1/^2 with *c* in [g/L], which corresponds to *B* = 1.06 ± 0.10, as calculated from eq 3. Assuming a helical conformation, instead of a wormlike chain fit at high *q*, we obtain *B* = 1.12 ± 0.10, which is effectively the same within experimental error. The *B*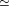 1 measured value, corresponding to an isotropic mesh of locally rigid rods, supports a locally stiff conformation, instead of a collapsed conformation or a large (strongly coiling) helix as previously suggested.^25^,^26^ Scaling theory also predicts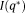 to vary as *c*^1/2^ in the semidilute regime, close to the observed dependence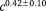.

Equations 1 and 3 give *B* = 1.06 and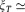 6Å for poor solvent and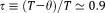, where *A* is calculated from Manning's theory^66^–^68^ and with *b* = 5.15Å, the monomer size. For good solvent, nearly identical values are obtained, indicating that both cases are compatible with our data. As cellulose is insoluble in water, we expect NaCMC to fall in the poor solvent case. The same calculation using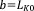, the Kuhn length (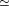 100Å), gives
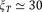
Å.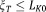 has the following physical interpretation: the chain is rigid up to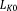 due to intrinsic stiffness and between *ξ_T_* and *ξ* due to electrostatic repulsion. This is equivalent to *B* = 1.

#### Comment on Clustering and Solubility

While our data are insufficient to resolve the structure of possible clusters (manifested by the low *q*-upturn), the high and intermediate *q* data allow us to effectively determine the solubility of NaCMC in water: the linear variation of *I*_0_ with
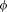
(1 − 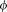) suggests no concentration dependent aggregation. Further, the values for the correlation length, in excellent agreement with the expected values for locally stiff chains, also indicate that no significant aggregation occurs; if a fraction of chains were aggregated, the number of effective chains contributing to the polyion mesh would decrease, leading to a higher *ξ*. Our results unambiguously establish the full solubility of NaCMC (D.S. = 1.2) in water at a molecular level. Further discussion of the low *q* upturn is provided in the Supporting Information.

#### Overlap Concentration

Estimating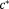 from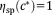 is commonly used,^42^ although the definition is somewhat arbitrary as it assumes *η*_sp_ = [*η*]*c* in the dilute regime, where [*η*] is the intrinsic viscosity, and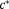 = 1/[*η*]. We obtain
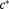
=0.07 g/L from this method, which we expect overestimates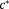 as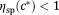 has been found experimentally for NaPSS.^69^ We may also estimate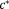 by equating the experimentally measured correlation length to the end-to-end distance (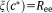) of chains at overlap and requiring that the chains occupy the whole volume of the solution (i.e.,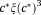 = 1, where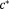 is in units of chains per unit volume), which is equivalent to eq 2 with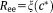. This method gives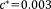 g/L. Calculating the volume fraction occupied by the chains (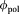) at *c* = 0.07 g/L assuming the end-to-end distance of the chains to be *ξ*(0.07 g/L)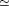 1120 Å (calculated using the experimental relation *ξ* = 296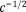), yields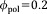, as opposed to the expected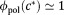, which appears to be a large difference. Repeating this calculation for a number of systems reported in the literature, we find the calculated volume fractions to range between 0.17 and 1, which is detailed in Supporting Information. Generally, we may expect that the calculation of the overlap from scattering would under predict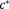 as it assumes chains to be rigid above *ξ_T_* in dilute solution when, in fact, chains in dilute solution are best described by a directed random walk of electrostatic blobs, and hence partially coiled. We can, therefore, regard the scattering and viscosity estimates of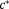 as lower and upper bounds, respectively.

### Rheology

#### Semidilute Unentangled Regime

The viscosity results for the unentangled region indicate a power law of 0.68 ± 0.02 with concentration, higher than the predicted slope from scaling theory of 0.5. This exponent does not appear to be universal for polyelectrolytes: NaPSS has been shown to scale as 0.35,^37^ poly(2-vinylpyrridine) quaternized with different ionic groups and to different extents shows an exponent of 0.5.^38^,^70^ Other polymers show greater exponents^36^,^71^ which arise, at least in some cases, due to shear thinning or residual salt.^37^ The higher 0.68 exponent may arise from intrinsic rigidity of the chain; scaling theory assumes that the total persistence length (*l*_p_) of chains is purely electrostatic and equal to the correlation length, that is, *l*_p_ = *l*_e_ = *ξ*, resulting in the end-to-end distance of the chain in semidilute solution varying as *R* ∼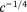. Considering the intrinsic rigidity of the chain (*l*_0_) results in a weaker variation of *R* with concentration, and should thus lead to a higher exponent in the viscosity (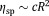 for Rouse chains).

**Figure 7:**
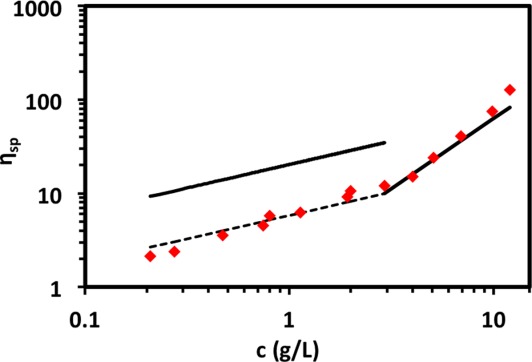
Viscosity in the semidilute unentangled and entangled regimes. Unentangled regime: the full line is computed from eq 4 with *B* =1 and *N* calculated using *M*_w_. The dotted line is a best fit line with exponent of 0.5, corresponding to *B* = 2.3. Entangled regime: the full line corresponds to eq 6 with *B* = 2.3 and *n* = 3.2. [Color figure can be viewed in the online issue, which is available at http://wileyonlinelibrary.com.]

Figure 7 shows the specific viscosity of semidilute unentangled solutions as a function of concentration. The solid line is the scaling prediction using eq 4 with *N* calculated from *M*_w_ and *B* = 1, which over-predicts the viscosity values by a factor of 3.5. The best fit to the data corresponds to *B* = 2.3, shown by the dashed line. Differences in *B* from SANS and rheology have been reported previously.^38^,^70^,^72^ We have analyzed data published by Kastner et al.^20^ and found *η*_sp_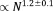 for unentangled salt free solutions of NaCMC in water of varying D.S. (0.75–1.47), in better agreement with the scaling prediction for flexible polyelectrolytes. We still expect the agreement between our data and eq 4 to be only qualitative as prefactors are ignored in its derivation. We, therefore, consider the *B* = 1.06 value from SANS to be a better estimate.

#### Semidilute Entangled and Concentrated Regimes

The terminal modulus may be estimated as *G*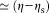/*τ*. We note, however, that *τ* (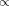 *N*^3^) is estimated from the onset of shear thinning and is likely to correspond to the high molecular weight fraction of the sample while the viscosity corresponds to the weight average of the entire distribution. For polydisperse systems, as is often the case of natural polymers, this will result in *G* being underestimated. We find *G* ∼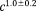 and about *k*_B_*T*/10 per chain, in disagreement with the scaling prediction of *k*_B_*T* per chain for unentangled solutions and of *G* ∼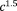 for semidilute entangled solutions. Power laws between 0.2 to 1.5 and prefactors close to *k*_B_*T* per chain have been found for various polyelectrolyte/solvent systems.^37^,^39^,^70^ Given the above considerations, it is likely the *k*_B_*T*/10 value arises due to sample polydispersity.

The scaling prediction for the slope in the entangled regime (3/2) is in good agreement with our best fit slope of 1.7 ± 0.2. The number of chain overlaps on the scale of the tube diameter needed to form an entanglement strand *n*, can be estimated from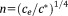.^33^ Using *c*_e_ = 2.9 g/L and our estimates of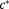, we calculate the number of entanglements per chain *n*, to be 5.5 using the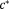 derived from scattering or *n* = 2.6 using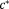 obtained from viscosity; we regard these as upper and lower bounds for *n*. Using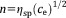,^33^ yields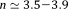. Leaving *n* as a free parameter in eq 6, a best fit to the data is obtained for *n* = 3.2, shown by the solid line in Figure 7. Equation 5 gives values approximately three times lower than eq 6 which can be expected due to the aforementioned polydispersity effect. This value may be compared with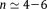 (obtained from the best fit lines in Fig. 4 of ref. 37 using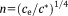 and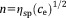) for NaPSS *N* = 3500, which has the same value of *Nb*/*B*, that is, the same effective contour length. NaCMC appears to require a similar number of chain overlaps to form an entanglement despite the large differences in molecular architecture.

The power law in the concentrated regime of 3.4 ± 0.2 is in agreement with the scaling prediction that concentrated polyelectrolyte solutions should behave similarly to concentrated neutral polymer solutions.

The crossover between the semidilute and concentrated regimes is predicted by scaling theory to happen when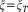, but that should not apply to more rigid polyelectrolytes. From rheology we conclude c_D_ = 14.4g/L, and we estimate from our SANS results, we estimate *ξ*(*c*_D_)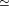 80 Å, much larger than the estimated values of *ξ_T_* and of the order of the intrinsic Kuhn length (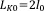) reported in the literature for NaCMC.^17^,^18^ *c*_D_ is also much lower than that of flexible polyelectrolytes such as NaPSS,^34^,^73^ or sodium polyacrylate^74^ (*c*_D_ ∼ 1 M,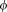 ∼ 0.3). Similarly, low concentrations have been reported for salt free xanthan or alginate solutions^43^,^75^ as well as poly (acrylamide-co-sodium 2-acrylamido-2-methylpropanesulfonate) with 10% degree of sulfonation.^71^ The correlation between *c*_D_ and *l*_0_ (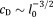) is further addressed in Supporting Information.

We do not observe a change from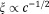 to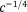 at *c*_D_, as reported in other systems,^45^,^73^ or a decrease in the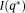 power law with concentration. The concentration c_D_ where entangled polyelectrolyte scaling of the viscosity gives way to concentrated solution is indicated by dashed lines in Figure 6 and the power laws persist for q* and *I*(q*) above and below c_D_.^73^ As the fraction of condensed counterions increases across *c*_D_ for NaCMC and other systems,^24^ we take the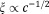 scaling as evidence that the electronstatic blob concept (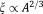 in poor solvent) does not apply here.

## CONCLUSIONS

We have studied aqueous solutions of carboxymethyl cellulose of average degree of substitution 1.2 in the semidilute and concentrated regimes by rheology and SANS. Both our SANS and rheology results indicate that NaCMC D.S. = 1.2 is molecularly dispersed in water. Rheologically, NaCMC shows similar behavior to other polyelectrolyte systems, with a near Fuoss law in the semidilute unentangled regime and in good agreement with scaling predictions in the entangled and concentrated regimes. Using SANS, we measure the cross sectional radius of the chain to be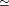 3.4 Å. At low concentrations, below
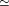
15 g/L, a shoulder appears in the scattering function at high *q*, the origin of which could not be resolved but appears to be associated to an intermolecular effect.

We measure the correlation length from the position of the peak in the scattering function, which is in good agreement with scaling predictions (*B* = 1.06,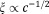). Interestingly, there is no change in the scaling of the peak position or intensity at the crossover to the concentrated regime, contrary to that observed in other systems. This contrasts with a sharp change in the viscosity scaling with concentration across c_D_. The crossover happens when *ξ*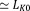 and cannot be rationalized in terms of the overlap of electrostatic blobs and correlation blobs, as predicted by scaling theory for flexible polyelectrolytes. Flexible polyelectrolyte scaling theory provides a good description of the concentration dependence of the viscosity, relaxation times, and correlation length for salt free aqueous NaCMC solutions.
