# Supplementary material for: Structure of Sodium Carboxymethyl Cellulose Aqueous Solutions: A SANS and Rheology Study
Source: J Polym Sci B Polym Phys. 2014 Dec 30;53(7):492–501. doi: 10.1002/polb.23657 (PMC4681322; doi:10.1002/polb.23657)
Supplement: Supplementary file 1 — Supplementary Information [file polb0053-0492-sd1.pdf]

# Supporting Information: Structure of Sodium Carboxymethyl Cellulose aqueous solutions: A SANS and rheology study.

Carlos G. Lopez, Sarah E. Rogers, Ralph H. Colby, Peter Graham, João T. Cabral

## 1 Scattering equations

The differential cross section of scattered intensity per unit volume in a small angle experiment for an n-component system may be written as:

$$I(q) = \rho \sum_{i=1}^{n} k_i k_j S_{ij}(q)$$

Where $\rho$ is the concentration in number of species per unit volume and $k_i = b_p$ - $b_s v_p / v_s$; $b$ is the coherent scattering length and $v$ is the partial molar volume. The subscript $p$ referrers to a solute species and $s$ refers to the solvent. Salt free polyelectrolyte solutions comprise three components: solvent, polymer and counter ions. $S(q)$ is often divided into an intramolecular part $P(q)$, the form factor and an intermolecular part, $H(q)$ where $q$ is the scattering wavevector, $S(q) \equiv P(q)H(q)$. While the form factor of polyelectrolytes in solution can be modelled by a semiflexible chain, the intermolecular structure factor $H(q)$ is significantly more complex to model. PRISM theory [1] has been used to successfully model data in selected systems and $q$ ranges. It does not predict the so-called low $q$ upturn, thought to be caused by multi chain domains, and is computationally demanding.

We follow a systematic approach to data fitting, building up from small length scales (high $q$) toward the forward scattering signal.

**High q: chain diameter**

At high q, we assume that no intermolecular effects are present and the single chain signal dominates [2, 3]. The form factor of a wormlike chain for q$l_p > 1$, where $l_p$ is the total persistence length of the chain, is given by the product of an infinitely long, thin wormlike chain $P_0 = \frac{\pi}{bq}$ and a term that accounts for its finite cross section ($P_{cs}$), here modelled a step function, corresponding to a cylinder of uniform density:

$$P_0 P_{cs}(q) \;=\; \frac{\pi}{bq}(\frac{2J_1(qr_p)}{qr_p})^2$$

where $J_1$ is a first order Bessel function of the first kind and $r_p$ defines the radius of the chain. The high $q$ data are therefore fitted with:

$$I(q)_{highq} = I_0/q(\frac{2J_1(qr_p)}{qr_p})^2 + S_{inc} \tag{1}$$

The constant $I_0$ is a function of the contrast factor, the polymer concentration and the monomer length (b = 5.15 Å) for a glucose unit. $S_{inc}$ accounts for the $q$-independent scattering.

**Intermediate q: correlation peak**

At lower $q$ values, inter molecular correlations become important and $H(q)$ is no longer negligible. Hammouda et al. [4] proposed a Lorentzian function to fit polyelectrolyte peaks and related the peak intensity to the solvation quality, by analogy with neutral polymers, where the random phase approximation establishes a direct link between the high $q$ intensity and the solvent quality. We find that, while a Lorentzian function can describe our data, it does so by forcing a constant slope at high $q$ by background fitting. If we use the value of $S_{inc}$ obtained by fitting a worm like chain to the high $q$ region of

our data, the Lorentzian fit becomes poor. We therefore opt to use an empirical function which correctly describes the peak profiles and is compatible with eq (1):

$$H(q) = \frac{1}{1 - exp(-(qd)^m - kq)} \tag{2}$$

which yields a descriptive fit to the data from which the $q^*$, $I(q^*)$, the peak position and intensity, and a sharpness parameter can be extracted. We assign no physical meaning to the fitting parameters m, d and k.

**Low $q$ upturn and fluctuations**

We model the upturn by adding a power law term:

$$I(q) = Dq^{-n} \tag{3}$$

where $D$ and $n$ are allowed to vary for each sample.

**Fitting procedure**

Data fitting was carried out by least squares minimisation using the error values estimated by Grasp or Mantid (the data reduction software for the D11/D22 and SANS2D beamlines respectively). Due to the high number of parameters in equations 1-3, the fitting is carried out sequentially. Equation 1 is fitted to the high q part of the data leaving $I_0$, $r_p$ and $S_{inc}$ as free parameters. Fitting different q ranges, always for $q \geq q^*$ we find greater consistency when fitting the data for $q \geq 1.5q^*$. The parameters $I_0$ and $S_{inc}$ vary linearly with $\phi$ and (1-$\phi$) respectively, where $\phi$ is the volume fraction occupied by the monomers. As explained in the main paper, a value of $r_p = 3.4$Å was selected and the data was refitted with this new value and $I_0$ and $S_{inc}$ left as free parameters. The new values of $I_0$ and $S_{inc}$ did not change significantly. The water content and $S_{inc}^{pol}$ were calculated using this last set of values. Next we fit the data around the peak position with equations 1 and 3

simultaneously allowing $I_0$ to vary (the value never deviates from the original by more $\simeq$ 5%). The term for the upturn is finally added and the full set of data is fitted leaving $D$, $n$, $d$, $m$, $k$ and $I_0$ free. The value of $n$ is found to be close to 3.6 for all samples and the previous step is repeated with $n$ fixed at 3.6.

**Fitting of high q data using a helical form factor**

We employ the 'Series of Coaxial Shifted Infinitely Long Thick Helices with a Uniform Electron Density' form factor from ref [5], set to $k = 1$, corresponding to the case of a single helix. A cylindrical helix is defined by $x = R^H cos(t)$, $y = R^H sin(t)$, $z = P/(2\pi)t$, where $R^H$ is the radius and $P$ is the pitch of the helix. Additionally, $r_p^H$, is the cross sectional radius of the helical chain. It is unclear to us the meaning of the pre-factor in of ref [5] expression as it is defined differently in two sections of the paper. In order to obtain the helical form factor in absolute units, we take the contour length of the helix to be $Nb$, and calculate the z-projected contour distance using arclength $= ((R^H)^2 + (P/2\pi)^2)^{1/2}t$, and require that the low q limit matches the form factor of a rod of equal mass per unit length, which is readily available in absolute units.

In the $q$-range studied, a number of combinations of $r_p^H$, $R^H$ and $P$ yield a form factor identical to that of a cylinder. Of course, a helix will reduce to a cylinder when $r_p^H$ or $P$ tend to infinity. We first consider the case of a thin helix, setting $r_p^H = 0$ and allowing $P$ to vary between 5 and 250Å, we obtain $R^H \simeq 2.5$Å and $P \geq 40$Å as valid fits. Setting $r_p^H = 2$, a more realistic value, and varying $P$ to vary over the same interval, we get $R \simeq 1 - 2$Å. While our data is insufficient to discriminate between a linear conformation and a helical one, we can state that our data is compatible with a linear conformation or a conformation with small helicity. The lateral fluctuations ($\approx R_H + r_p^H \simeq 3.5 \pm 1.0$) are within the cylindrical fit value of $r_p \simeq 3.4 \pm 1.0$Å. The mass per unit length being $\simeq 5\%$ higher than the straight conformation case. The water content calculated from the helical

form factor is $\simeq 4\%$ lower than for the straight chain.

**Low q upturn**

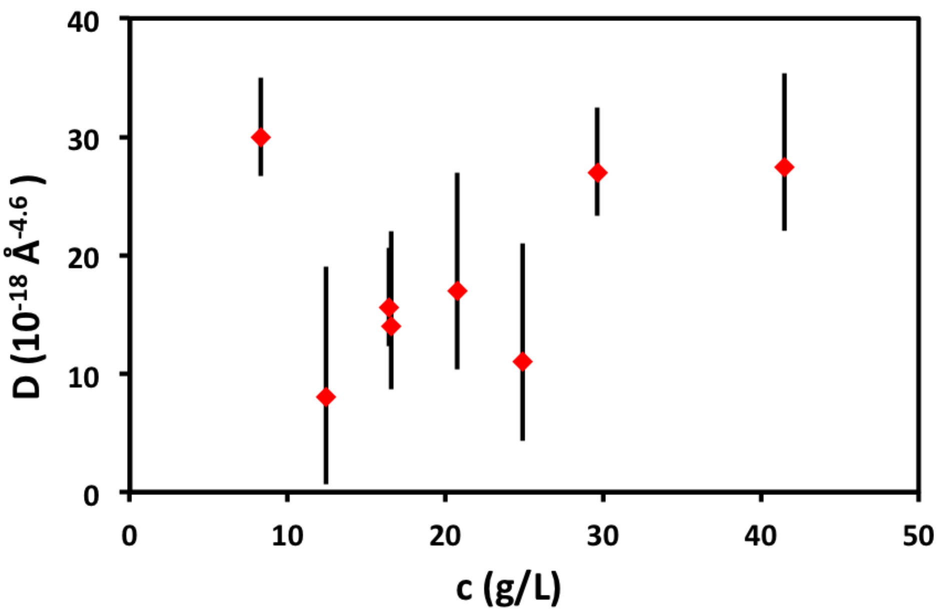


Figure 1: Low-q upturn intensity $D$ from power law fits $I(q) = D/q^{3.6}$ at low $q$ as a function of concentration.

*Low q upturn intensity.* The upturn parameter $D$ is plotted as a function of concentration in Fig. 1. We lack the $q$ range or statistics in the low $q$ region for a number of samples to estimate $D$, and therefore only plot $D$ for concentrations at which it can be obtained with reasonable accuracy. Although it is not possible to deduce a clear functional dependence for the upturn intensity from our data, we find that $D$ broadly increases with $c$. We note that all measurements are in the concentrated regime except for the 8 g/L sample which is in the semidilute region.

## 2 Solution preparation and time dependent effects

Figure 1 shows the peak position $q^*$ of NaCMC (D.S.=1.2) in $D_2O$, measured by SANS, as a function of concentration. Different symbols correspond to approximate times after sample preparation. No trend with time can be observed, indicating that the same polymer fraction is dissolved at all measurement timescales. In addition, we have found that $c^{1/2}$ dependence applies across the whole concentration range and that the solvent quality parameter $B = 1$, indicating that $q^*$ corresponds to a mesh of isotropic, locally stiff polymer segments. These observations collectively establish that NaCMC D.S. = 1.2 is molecularly soluble in water within this concentration range.

The viscosity measurements were carried out between 1 and 4 days after sample preparation. We evaluated a possible time dependence in the viscosity by measuring one sample c $\simeq$ 25g/L using a DVI-Prime Brookfield viscometer with a Couette geometry. We found that the viscosity decreases by about 2% from day one to day 2 and by a further 2% by day 4. Approximately 10 months after sample preparation, the viscosity decreased between 20 and 30% for several samples tested. The time dependence of the viscosity may be described by $\eta_{sp} \simeq$ -12$ln(t)$ + 358, where $t$ is the time in days, for this time window. This slow dependence is likely due to small changes in the molecular weight caused by polymer degradation over time. The results are plotted in Fig 2.

The pH of solutions in the 5-35g/L concentration range was $\simeq$ 7.5-8 on fresh samples and showed no measurable time evolution on the 10 month samples.

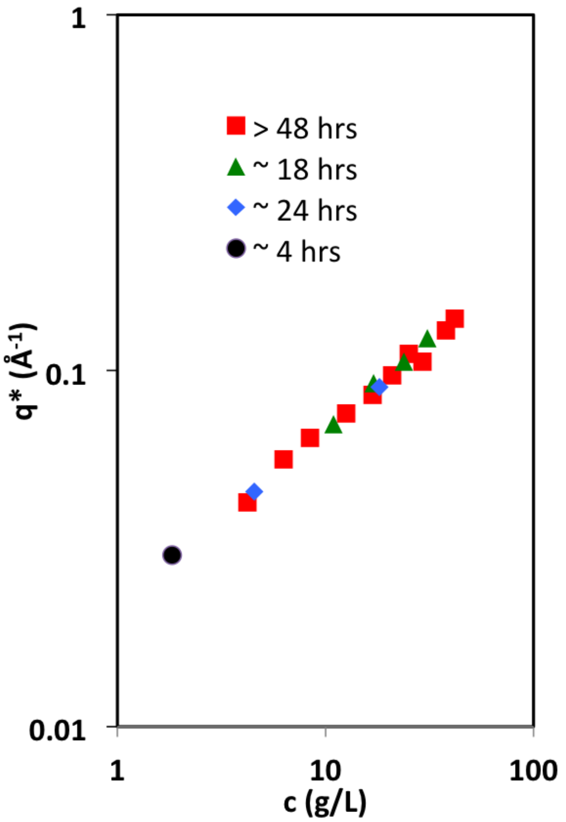


Figure 2: Correlation peak position q* ($=2\pi/\xi$, where $\xi$ is correlation length) as a function of NaCMC (D.S.=1.2) in $D_2O$ concentration measured at different times after sample preparation. No time dependent effects are observed, as shown by the well defined $c^{1/2}$ behaviour obeyed by all samples.

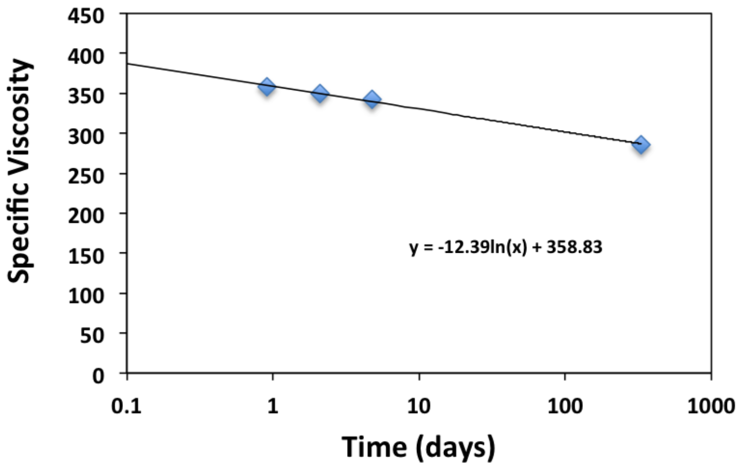


Figure 3: Specific viscosity as a function of time from sample preparation for samples c=25 g/L NaCMC in water. The last point was estimated as 75% of the viscosity at day 1. Logarithmic line serves as a guide the eye.

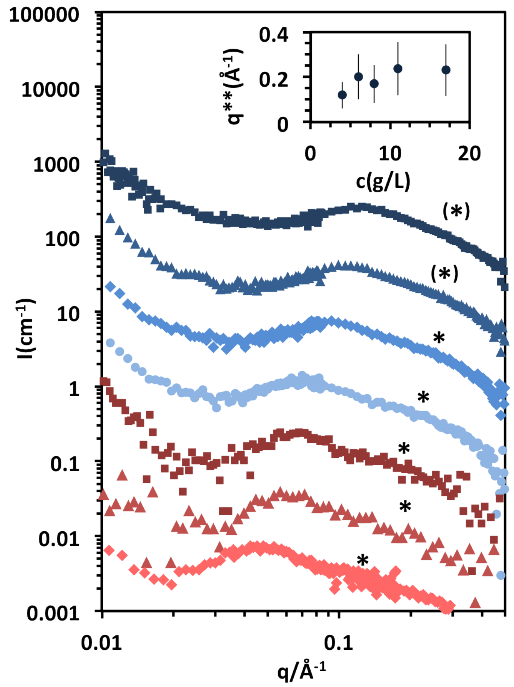


Figure 4: SANS profiles of NaCMC in $D_2O$ as a function of concentration, $*$ symbols denote the approximate position of the shoulder ($q^{**}$) observed at high q. Inset shows the values of $q^{**}$ as a function of concentration. Concentrations, acquisition times and beamlines, from bottom to top: 4(15 min high q, 6 min mid q, D22) , 6 (38 min, SANS2D), 8 (38 min, SANS2D), 11, 17, 23, 30g/L (3 min high q, 5 min mid q, D11) .

Figure 3 shows the SANS profiles of NaCMC as a function of concentration. The $*$ symbols denote the approximate position of the shoulder observed at high q. Due to low sample count rates, and thus low statistics, it is difficult to accurately resolve the position of the shoulder. The approximate wavenumber $q^{**}$ is shown in the inset as a function of concentration. At sufficiently high concentrations, the shoulder can no longer be resolved (position indicated with a $(*)$, not included in the inset) and the scattering signal is fully described by the cylinder form factor. Despite the large experimental uncertainty, there is a weak positive concentration dependence, which could support the assignment of this

feature to an intermolecular effect (instead of a form factor). The determination of such structure factor is however not possible from our scattering data of such weakly scattering solutions.

# 3 Crossover concentrations, comparison with literature data.

## 3.1 Overlap concentration

We noted in the paper that a significant disparity exists between the overlap concentration calculated from $\eta_{sp}(c^*) = 1$ method, denoted $c_v^*$, and from solving eq (2) for $c$ in main paper with $R_{ee} = \xi(c)$, denoted $c_s^*$. The $\xi(c)$ relationships are obtained from SANS data. The table below shows the values for $c^*$ obtained a number of systems using these two methods. The calculated volume fraction occupied by the chains at $c_v^*$ ($\phi_{pol}^*$) assuming the end to end distance of the chains is given by $R_{ee} = \xi(c_v^*)$ and $\phi_{pol}^* = c_v^* R_{ee}^3$ where c is in units of number of chains per unit volume.

| Polyelectrolyte | $M_w$ (kg/mol) | $c_s^*$ (g/L) | $c_v^*$ (g/L) | $\phi_{pol}^*$ | ref |
|---|---|---|---|---|---|
| NaPAMS N = 300 | 70 | 3.37 | 3.22 | 1.02 | [6] |
| poly(diisobutylene-alt-maleate) | 82 | 0.39 | 0.51 | 0.88 | [7] |
| NaPSS [a] | 200 | 0.11 | 0.34 | 0.56 | [8] |
| NaCMC | 280 | 0.003 | 0.07 | 0.2 | This work |
| poly(styrene-alt-maleate) | 350 | 0.031 | 0.128 | 0.5 | [7] |
| PMVP-Cl-55 | 454 | 0.032 | 0.23 | 0.36 | [9, 10] |
| NaPAMS N = 2200 | 500 | 0.066 | 0.069 | 0.98 | [6] |
| HA | 1260 | 0.00046 | 0.016 | 0.17 | [11, 12] |

Table 1: Polyelectrolyte, $M_w$, $c_v^*$, $c_s^*$ and $\phi^*$. and All refer to aqueous systems except PMVP-Cl-55 for which the solvent is ethylene glycol. The reference column includes the source for $\eta_{sp}(c^*) = 1$ and $\xi(c)$. [a] taken from line of best fit to a wide range of $M_w$ in ref [8].

It thus appears that the discrepancy between the two methods is common to most

systems and our result is within the typical range of reported values.

### 3.2 Crossover to the concentrated, correlation with intrinsic flexibility

We observe that the value of the correlation length at the crossover to the concentrated regime ($\xi(c_D)$) is much larger than the thermal blob size ($\xi_T$) and of the order of the intrinsic Kuhn length ($L_{K0}$) of NaCMC. Scaling theory assumes that polyelectrolytes are flexible above the monomer size and hence are collapsed or extended rather than rod-like at distances smaller than $\xi_T$. With respect to the intrinsic persistence length $l_0 = L_{K0}/2$, it is often the case that $\xi_T \leq l_0$, and in this situation we could expect polyelectrolytes to remain rigid at small length scales, if the covalent bonds or steric hinderance that give rise to intrinsic rigidity involve energies much greater than $k_BT$. A crossover may then be expected at $\xi = l_0$, rather than $\xi = \xi_T$. In this section, we compile literature data on other polyelectrolyte systems to asses whether this conjecture is consistent for polyelectrolytes of different persistence lengths.

**Literature data**

Table 1 shows the intrinsic persistence length, $c_D$ in molar units and the correlation length at $c_D$ for a number of polyelectrolyte systems, along with the method used to estimate the crossover to the concentrated regime. For the majority of these systems, the crossover is identified from rheological methods, while for the first three it is identified from scattering measurements of the correlation length, where a change of the scaling of $\xi$ with concentration from 0.5 to 0.25 is observed. For NaPSS, the crossover has been identified by scattering techniques (SANS and SAXS) and osmometry, all agreeing within experimental error. $\xi(c_D)$ is obtained by extrapolating scattering measurements from the semidilute regime to $c_D$ in the case of poly(2-vinylpyridine-co-N-methyl-2-vinylpyridinium chloride) (PMVP-Cl-55) and sodium polyacrylate (NaPA), and from the concentrated back

to $c_D$ for xanthan and alginate. $c_D$ and $\xi(c_D)$ for hyaluronic acid (HA) are obtained from the results of Lorchat [11] et al. who observed a power law change in the scaling of $\xi$ from 1/2 to 1/4. Using SAXS Salamon et al.[13] however see no crossover in the scaling of the correlation length with concentration up to 0.2M for sodium hyalorunate. The case of xanthan is somewhat complex [14] as it adopts a double helix conformation in solution. The power law in the concentrated regime is $\simeq 0.5$ at high concentrations and $\simeq 0.3$ at lower concentrations (but still in the concentrated regime as identified by rheology). For systems where no data exists for the intrinsic persistence length: PMVP-Cl, sodium poly($\alpha$methyl styrene sulfonate) (NaP$\alpha$MSS) and polydiallyldimethylammonium chloride (PDADMAC), the persistence length of the neutral polymer has been used.

| Polyelectrolyte | $l_0$ / Å | $c_D$/M | $\xi(c_D)$/Å | Method | ref |
|---|---|---|---|---|---|
| NaP$\alpha$MSS | 22 | 0.5 | 52 | SANS | [15] |
| PDADMAC | 26 | 0.15 | 79 | SANS | [11, 15]. |
| NaPSS | 9.5 | 1 | 35 | SANS, SAXS, Osmometry | [15–19] |
| NaCMC | 50 | 0.054 | 84 | Rheology | This work [20, 21] |
| PMVP-Cl-55 | 15 | 1 | 43 | Rheology | [9, 10, 22–24] |
| NaPa | 15 | 1.3 | 32 | Rheology | [25–27] |
| Alginate | 103 | 0.07 | 139 | Rheology | [28–33] |
| HA | 65 | 0.08 | 71 | SAXS | [11, 34–37] |
| Xanthan | 930 | 0.002 | 314 | Rheology | [14, 38–41] |
| Chitosan | 76 | | | | [42–44] |
| PPP[a] | 200 | | | | [45–47] |
| DNA | 550 | | | | [48, 49] |

Table 2: Polyelectrolyte, intrinsic persistence length, correlation length at $c = c_D$ and method(s) for estimating $c_D$. All refer to aqueous systems except QP2VP-55 for which the solvent is ethylene glycol. The reference column includes the source for $c_D$, $\xi$ and $l_0$. For the latter quantity, a range of values can be found, whose spread is used to compute the error bars in the two figures below. [a] indicates polyelectrolytes with a poly(p-phenylene) backbone.

The figures below show $c_D$ and $\xi(c_D)$ as a function of $l_0$. The former shows a clear

correlation, with intrinsically stiffer polyelectrolytes having a lower $c_D$. The opposite trend is observed between $l_0$ and $\xi(c_D)$. We may expect no correlation for the more flexible systems where the concept of a thermal blob applies. The dashed line indicates $\xi(c_D) = 10.5 l_0^{1/2}$ while the full line indicates $\xi(c_D) = l_0$. The former shows a better agreement suggesting that, while there is a correlation between $l_0$ and $\xi(c_D)$, it is not linear, and therefore the crossover to the concentrated is unlikely to correspond to a crossover between the two quantities.

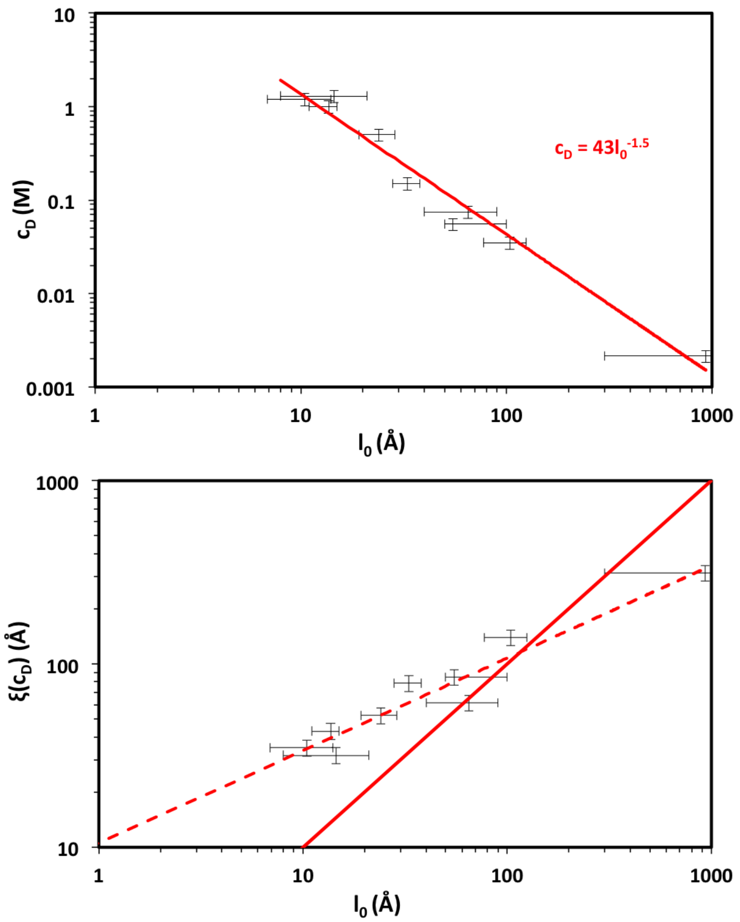


Figure 5: (Top): $c_D$ vs. $l_0$ for the systems listed in Table 1, line is a power law best fit $c_D = 43l_0^{-3/2}$. (Bottom): Correlation length at the crossover to the concentrated ($\xi(c_D)$) as identified by scattering or rheology (see text) vs. intrinsic persistence length $l_0$. Dashed line indicates is a power law best fit $\xi(c_D) = \sqrt{110l_0}$, full line indicates $\xi(c_D) = 2.5l_0$.

*Power law dependence of the correlation length with concentration, flexible vs. stiff polyelectrolytes*

NaPSS, NaP$\alpha$MSS, PDADMAC and HA show a change in the scaling of the correlation length with concentration from -0.5 to -0.25 at $\xi(c_D)$ while NaCMC and NaHA do not. Additionally, $\xi$ for DNA and chitosan scales as $c^{1/2}$ even when $\xi \leq l_0$. It would appear therefore that the scaling of the correlation length remains unchanged for stiff polyelectrolytes but not for flexible ones. One exception to this is HA and NaHA, where, despite having the same intrinsic flexibility, the former does not show a change in the scaling while the latter does, hence, there must be other parameters affecting whether this change in scaling happens or not.

## 4 Effect of intrinsic flexibility on unentangled viscosity

We next consider how the rheology of semiflexible polyelectrolytes may differ from that of flexible ones. The specific viscosity of polyelectrolytes in the semidilute unentangled regime can be described as:

$$\eta_{sp} = Kc^{\beta} \tag{4}$$

The best fit to our data is found for K = 6 and $\beta = 0.68$. Equation (4), commonly used to calculate B from viscosity data [9, 50], which predicts $\beta = 0.5$ uses two simplifications: firstly, it does not include pre factors in the calculation of the Rouse time and Zimm times; secondly, it assumes the step length (or persistence length $l_p$) is purely electrostatic and equal to the correlation length, that is $l_p = l_e = \xi$.

Including the pre-factors in the Rouse and Zimm times affects $K$, but does not change the exponent $\beta$. We expect the agreement of $K$ with the data to be qualitative, as it

involves a number of parameters such as the friction factor, for which we do not have a precise form. We may however expect the scaling prediction for $\beta$ to be more accurate, given the close match between theory and experiment for neutral polymer solutions and some flexible polyelectrolyte systems [51].

In contrast with the scaling assumption $l_p = \xi$, for semiflexible polyelectrolytes, we should take into account the intrinsic rigidity of the chain. It is also important to note that neutron scattering experiments on NaPSS show that the electrostatic persistence length is approximately proportional but not equal to the correlation length. Spiteri [19] finds $l_e \simeq 0.6\xi$, in agreement with theoretical predictions that $l_e \propto \kappa^{-1}$. It is of course possible for $l_e$ to have a more complex concentration dependence. For simplicity here we write:

$$l_p = l_0 + \gamma\xi \tag{5}$$

Davis [20] calculated the electrostatic persistence length of NaCMC using non linear electrostatic wormlike theory [52] for dilute solutions of different ionic strengths in the range 0.01-0.2M. Assuming the persistence length is just a function of the total ionic strength and extrapolating his values to our concentration range, we obtain $\gamma = 0.54$. We may expect this to be slightly lower given the assumption of Manning condensation and for our semidilute solutions, the charge density is lower than this estimate. We now revise the resulting exponent taking into account the intrinsic rigidity.

**Free draining ideal chain**

Assuming the chain to be free draining, the Rouse model applies, and the viscosity is then given by:

$$\eta_{sp} = \frac{\pi}{36} c N_l \zeta R^2 \tag{6}$$

where R is the end-to-end distance of the chain given by $R^2 = N_l l_p^2$, $l_p$ is the step length, $N_l$ is the number of steps per chain ($L/l_p$) and $\zeta$ is the friction coefficient of a segment, which can be modelled by that of a sphere: $\zeta = 6\pi\eta_s l_p$. Combining Eqs (1) and (2), we obtain $\beta = 0.61$, which compares more favourably with the experimentally measured $\beta = 0.68$ obtained for $\gamma = 0.26$.

The departure from the Fuoss exponent might be due to the intrinsic rigid of the chain, which results in a power law dependence of the step length smaller than 0.5, and in turn in a variation of the end to end distance of $R \sim c^{-1/5}$, instead of the -1/4 dependence for flexible polyelectrolytes.

**Rouse-Zimm chain**

If we assume the chain is non draining up to the segment length and free draining above it, we can calculate the Zimm time of a segment ($\tau_Z \sim l_p^3$) and estimate the total relaxation time of the chain to be $\tau = \tau_Z(\frac{Nb}{l_p})^2$. The viscosity is then estimated by multiplying the relaxation time by the modulus ($k_B$T per chain). This gives an exponent of $\beta = 0.61$, identical to the free draining case. The same chain conformation but assuming chains to be non draining up to the correlation length (instead of $l_p$), yields $\beta = 0.72$.

For all the previous calculations, if the intrinsic persistence length is set to zero, we trivially recover the Fuoss scaling of 0.5. We do not know the exact length scale of hydrodynamic screening, which is required for an accurate calculation of the viscosity. However, we find that for chains with some intrinsic rigidity, we can expect the power law in the unentangled regime to be higher those of flexible polyelectrolytes. Taking $l_0 = 54$ Å and $\gamma = 0.54$, depending on the model used, the exponent is expected to be between 0.61 and 0.72, in reasonable agreement with the observed $0.68 \pm 0.02$.

## References

[1] Arun Yethiraj. Liquid state theory of polyelectrolyte solutions. *J. Phys. Chem. B*, 113(6):1539–1551, 2009.

[2] K. Nishida, K. Kaji, T. Kanaya, and T. Shibano. Added salt effect on the intermolecular correlation in flexible polyelectrolyte solutions: small-angle scattering study. *Macromolecules*, 35(10):4084–4089, 2002.

[3] K. Kassapidou, W. Jesse, M. E. Kuil, A. Lapp, S. Egelhaaf, and J. R. C. van der Maarel. Structure and charge distribution in dna and poly(styrenesulfonate) aqueous solutions. *Macromolecules*, 30(9):2671–2684, 1997.

[4] Boualem Hammouda, Ferenc Horkay, and Matthew L. Becker. Clustering and solvation in poly(acrylic acid) polyelectrolyte solutions. *Macromolecules*, 38(5):2019–2021, 2005.

[5] Pablo Székely, Avi Ginsburg, Tal Ben-Nun, and Uri Raviv. Solution x-ray scattering form factors of supramolecular self-assembled structures. *Langmuir*, 26(16):13110–13129, 2010.

[6] Wendy E. Krause, Julia S. Tan, and Ralph H. Colby. Semidilute solution rheology of polyelectrolytes with no added salt. *J. Polym. Sci., Part B: Polym. Phys.*, 37(24):3429–3437, 1999.

[7] E. Di Cola, N. Plucktaveesak, T. A. Waigh, R. H. Colby, J. S. Tan, W. Pyckhout-Hintzen, and R. K. Heenan. Structure and dynamics in aqueous solutions of amphiphilic sodium maleate-containing alternating copolymers. *Macromolecules*, 37(22):8457–8465, 2004.

[8] David C. Boris and Ralph H. Colby. Rheology of sulfonated polystyrene solutions. *Macromolecules*, 31(17):5746–5755, 1998.

[9] Shichen Dou and Ralph H. Colby. Charge density effects in salt-free polyelectrolyte solution rheology. *J. Polym. Sci., Part B: Polym. Phys.*, 44(14):2001–2013, 2006.

[10] Brett D. Ermi and Eric J. Amis. Domain structures in low ionic strength polyelectrolyte solutions. *Macromolecules*, 31(21):7378–7384, 1998.

[11] Philippe Lorchat, Iuliia Konko, Jérôme Combet, Jacques Jestin, Albert Johner, André Laschewski, Sergei Obukhov, and Michel Rawiso. New regime in polyelectrolyte solutions. *EPL (Europhysics Letters)*, 106(2):28003, 2014.

[12] Tu Luan Zinan Zhang Hongbin Zhang Fengyuan Yu, Fei Zhang. Rheological studies of hyaluronan solutions based on the scaling law and constitutive models. *Polymer*, 55(1):295–301, 2014.

[13] K. Salamon, D. Aumiler, G. Pabst, and T. Vuletia. Probing the mesh formed by the semirigid polyelectrolytes. *Macromolecules*, 46(3):1107–1118, 2013.

[14] Michel Milas, Marguerite Rinaudo, Robert Duplessix, Redouane Borsali, and Peter Lindner. Small angle neutron scattering from polyelectrolyte solutions: From disordered to ordered xanthan chain conformation. *Macromolecules*, 28(9):3119–3124, 1995.

[15] P. Lorchat. Thesis, institut charles sadron - strasbourg , 2012.

[16] K. Nishida, K. Kaji, and T. Kanaya. High concentration crossovers of polyelectrolyte solutions. *J. Chem. Phys.*, 114(19):8671–8677, 2001.

[17] Lixiao Wang and Victor A. Bloomfield. Osmotic pressure of polyelectrolytes without added salt. *Macromolecules*, 23(3):804–809, 1990.

[18] Eigo Hirose, Yoshimi Iwamoto, and Takashi Norisuye. Chain stiffness and excluded-volume effects in sodium poly (styrenesulfonate) solutions at high ionic strength. *Macromolecules*, 32(25):8629–8634, 1999.

[19] M.N. Spiteri. Thesis, universite orsay - paris-sud, 1997.

[20] Richey M. Davis. Analysis of dilute solutions of (carboxymethyl)cellulose with the electrostatic wormlike chain theory. *Macromolecules*, 24(5):1149–1155, 1991.

[21] Kenji Kamide, Masatoshi Saito, and Hidemastu Suzuki. Persistence length of cellulose and cellulose derivatives in solution. *Makromol Chem Rapid Commum.*, 4(1):33–39, 1983.

[22] Patrick Knappe, Ralf Bienert, Steffen Weidner, and Andreas F. Thnemann. Characterization of poly(n-vinyl-2-pyrrolidone)s with broad size distributions. *Polymer*, 51(8):1723 – 1727, 2010.

[23] Georges M Pavlov, Evguenij F Panarin, Evgueniya V Korneeva, Constantin V Kurochkin, Vadim E Baikov, and Vera N Ushakova. Hydrodynamic properties of poly (1-vinyl-2-pyrrolidone) molecules in dilute solution. *Makromolekulare Chemie*, 191(12):2889–2899, 1990.

[24] David P Norwood, Edson Minatti, and Wayne F Reed. Surfactant/polymer assemblies. 1. surfactant binding properties. *Macromolecules*, 31(9):2957–2965, 1998.

[25] Ralf Schweins, Jutta Hollmann, and Klaus Huber. Dilute solution behaviour of sodium polyacrylate chains in aqueous nacl solutions. *Polymer*, 44(23):7131–7141, 2003.

[26] F Bordi, RH Colby, C Cametti, L De Lorenzo, and T Gili. Electrical conductivity of polyelectrolyte solutions in the semidilute and concentrated regime: the role of

counterion condensation. *The Journal of Physical Chemistry B*, 106(27):6887–6893, 2002.

[27] Yoshio Muroga, Ichiro Noda, and Mitsuru Nagasawa. Investigation of local conformations of polyelectrolytes in aqueous solution by small-angle x-ray scattering. 1. local conformations of poly (sodium acrylates). *Macromolecules*, 18(8):1576–1579, 1985.

[28] Simina Popa-Nita, Cyrille Rochas, Laurent David, and Alain Domard. Structure of natural polyelectrolyte solutions: Role of the hydrophilic/hydrophobic interaction balance. *Langmuir*, 25(11):6460–6468, 2009.

[29] Cristina Rodríguez-Rivero, Loic Hilliou, Eva M Martín del Valle, and Miguel A Galán. Rheological characterization of commercial highly viscous alginate solutions in shear and extensional flows. *Rheologica Acta*, 53(7):559–570, 2014.

[30] Olav Smidsrød. Solution properties of alginate. *Carbohydrate research*, 13(3):359–372, 1970.

[31] F Baños, Ana I Díez Peña, J Ginés Hernánez Cifre, M Carmen López Martínez, Alvaro Ortega, and José García de la Torre. Influence of ionic strength on the flexibility of alginate studied by size exclusion chromatography. *Carbohydrate polymers*, 102:223–230, 2014.

[32] Hucheng Zhang, Hanqing Wang, Jianji Wang, Ruifang Guo, and Qingzhi Zhang. The effect of ionic strength on the viscosity of sodium alginate solution. *Polymers for Advanced Technologies*, 12(11-12):740–745, 2001.

[33] M Rinaudo and D Graebling. On the viscosity of sodium alginates in the presence of external salt. *Polymer Bulletin*, 15(3):253–256, 1986.

[34] E Buhler and F Boué. Persistence length for a model semirigid polyelectrolyte as seen by small angle neutron scattering: a relevant variation of the lower bound with ionic strength. *The European Physical Journal E: Soft Matter and Biological Physics*, 10(2):89–92, 2003.

[35] Mary K Cowman and Shiro Matsuoka. Experimental approaches to hyaluronan structure. *Carbohydrate research*, 340(5):791–809, 2005.

[36] Kikuko Tsutsumi and Takashi Norisuye. Excluded-volume effects in sodium hyaluronate solutions revisited. *Polymer journal*, 30(4):345–349, 1998.

[37] Claude Oelschlaeger, M Cota Pinto Coelho, and Norbert Willenbacher. Chain flexibility and dynamics of polysaccharide hyaluronan in entangled solutions: a high frequency rheology and diffusing wave spectroscopy study. *Biomacromolecules*, 14(10):3689–3696, 2013.

[38] Nicholas B. Wyatt and Matthew W. Liberatore. Rheology and viscosity scaling of the polyelectrolyte xanthan gum. *J. Appl. Polym. Sci.*, 114(6):4076–4084, 2009.

[39] Catherine Esquenet and Eric Buhler. Aggregation behavior in semidilute rigid and semirigid polysaccharide solutions. *Macromolecules*, 35(9):3708–3716, 2002.

[40] Gisela Berth, Herbert Dautzenberg, Bjørn E Christensen, Stephen E Harding, Gudrun Rother, and Olav Smidsrød. Static light scattering studies on xanthan in aqueous solutions. *Macromolecules*, 29(10):3491–3498, 1996.

[41] B Tinland and M Rinaudo. Dependence of the stiffness of the xanthan chain on the external salt concentration. *Macromolecules*, 22(4):1863–1865, 1989.

[42] Marguerite Rinaudo, Michel Milas, and Pham Le Dung. Characterization of chitosan.

influence of ionic strength and degree of acetylation on chain expansion. *International Journal of Biological Macromolecules*, 15(5):281–285, 1993.

[43] Jaepyoung Cho, Marie-Claude Heuzey, André Bégin, and Pierre J Carreau. Viscoelastic properties of chitosan solutions: Effect of concentration and ionic strength. *Journal of Food Engineering*, 74(4):500–515, 2006.

[44] Gisela Berth, Herbert Dautzenberg, and Martin G Peter. Physico-chemical characterization of chitosans varying in degree of acetylation. *Carbohydrate Polymers*, 36(2):205–216, 1998.

[45] S Vanhee, R Rulkens, U Lehmann, Ch Rosenauer, M Schulze, W Köhler, and G Wegner. Synthesis and characterization of rigid rod poly (p-phenylenes). *Macromolecules*, 29(15):5136–5142, 1996.

[46] P. Galda. Thesis, karlsruhe, 1994.

[47] BL Farmer, BR Chapman, DS Dudis, and WW Adams. Molecular dynamics of rigid rod polymers. *Polymer*, 34(8):1588–1601, 1993.

[48] Jamie E Godfrey and Henryk Eisenberg. The flexibility of low molecular weight double-stranded dna as a function of length: Ii. light scattering measurements and the estimation of persistence lengths from light scattering, sedimentation and viscosity. *Biophysical chemistry*, 5(3):301–318, 1976.

[49] Paul J Hagerman. Investigation of the flexibility of dna using transient electric birefringence. *Biopolymers*, 20(7):1503–1535, 1981.

[50] Shichen Dou and Ralph H. Colby. Solution rheology of a strongly charged polyelectrolyte in good solvent. *Macromolecules*, 41(17):6505–6510, 2008.

[51] Ralph H. Colby. Structure and linear viscoelasticity of flexible polymer solutions: comparison of polyelectrolyte and neutral polymer solutions. *Rheol. Acta*, 49(5):425–442, 2010.

[52] R. M. Davis and W. B. Russel. On the theory of dilute polyelectrolyte solutions: Extensions, refinements, and experimental tests. *J. Polym. Sci. B Polym. Phys.*, 24(3):511–533, 1986.
